# NDRG2 contributes to cisplatin sensitivity through modulation of BAK-to-Mcl-1 ratio

**DOI:** 10.1038/s41419-017-0184-3

**Published:** 2018-01-18

**Authors:** Soojong Park, Sang-Seok Oh, Ki Won Lee, Yeon-Kyeong Lee, Nae Yu Kim, Joo Heon Kim, Jiyun Yoo, Kwang Dong Kim

**Affiliations:** 1 Division of Applied Life Science (BK21 Plus), Gyeongsang National University, Jinju, 52828 Republic of Korea; 2 Department of Internal Medicine, Eulji University School of Medicine, Daejeon, 35233 Republic of Korea; 3 Department of Pathology, Eulji University School of Medicine, Daejeon, 35233 Republic of Korea; 4 Division of Life Science, Gyeongsang National University, Jinju, 52828 Republic of Korea; 5 PMBBRC, Gyeongsang National University, Jinju, 52828 Republic of Korea

## Abstract

The downregulation *of N*-*Myc downstream*-*regulated gene 2* (*NDRG2*) is known to be associated with the progression and poor prognosis of several cancers. Sensitivity to anti-cancer may be associated with a good prognosis in cancer patients, and NDRG2, which is induced by p53, sensitizes the cells to chemotherapy. However, the unique function of NDRG2 as an inducer of apoptosis under chemotreatment has not been sufficiently studied. In this study, we investigated the role of NDRG2 in chemo-sensitivity, focusing on cisplatin in U937 histiocytic lymphoma, which has the loss-of-functional mutation in p53. NDRG2 promoted the sensitivity to cisplatin through the modulation of the BAK-to-Mcl-1 ratio. The degradation of Mcl-1 and increase in BAK were mediated by JNK activation and the eIF2α/p-eIF2α pathway, respectively, which depended on PKR activation in NDRG2-overexpressed U937 (U937-NDRG2) cells. NOX5 was highly expressed in U937-NDRG2 cells and contributed to ROS production after cisplatin treatment. ROS scavenging or NOX5-knockdown successfully inhibited the sensitivity of U937-NDRG2 cells to cisplatin. Taken together, these findings indicate that NDRG2 contributed to the increased sensitivity to ciplatin through the modulation of Bak-to-Mcl-1 ratio regulated by NOX5-ROS-PKR pathway; therefore, we suggest that NDRG2 may be a molecular target for improving the efficacy of drug treatment in cancer patients.

## Introduction

*N-Myc downstream-regulated gene 2* (*NDRG2*) has been reported to be tumor suppressor that is downregulated in aggressive tumor tissues and associated with the progression and prognosis of diverse cancers^1^. Several studies have suggested that NDRG2 plays an important role in tumor suppression, including the inhibition of tumor cell proliferation, invasion, and metastasis, in several cancer cell lines^2–6^.

A representative tumor suppressor, p53, plays a key role in apoptosis associated with DNA damage induced by anti-cancer drugs, such as cisplatin^7^. P53 induces the expression of pro-apoptotic proteins, such as Bax^8^, Noxa^9^ and Puma^10^, and downregulates the level of Bcl-2^11^. Recently, it has been demonstrated that NDRG2 plays a role in apoptosis induction, with a high number of apoptotic cells observed among NDRG2-expressed carcinoma cells compared with those among mock cells under metabolic stress or treatment anti-cancer drugs^1,3,12,13^. NDRG2 mRNA and protein expression are induced by p53 in tumor cells, and the silencing of NDRG2 attenuates p53-mediated apoptosis^14^. NDRG2 was suggested as a p53-inducible target and an effecter protein in p53-mediated apoptosis^15,16^. Conversely, knockdown of NDRG2 sensitized cervical cancer Hela cells, which have a mechanism inactivating p53 induced by E6 viral oncoprotein of HPV^17–19^, to cisplatin through suppressing Bcl-2 expression^20^. However, the molecular mechanism underlying the function of NDRG2 on apoptosis has not been sufficiently analyzed. Furthermore, whether NDRG2 has a pro-apoptotic function when p53 is active remains unclear.

In this study, we investigated the unique function of NDRG2 associated with drug-induced apoptosis, using an NDRG2-negative U937 cell line having a loss-of-function mutation in p53^21^. This made it possible to examine the unique function of NDRG2 in cisplatin-mediated apoptosis by excluding the function of p53. We established NDRG2-overexpressing U937 (U937-NDRG2), which exhibited higher sensitivity to ciplatin compared with mock cells (U937-Mock). We investigated how NDRG2 contributed to the increased sensitivity to cisplatin based on the cellular signaling events that induced apoptosis.

## Results

### NDRG2 made U937 cells more sensitive to cisplatin through the modulation of the BAK-to-Mcl-1 ratio

**Fig. 1:**
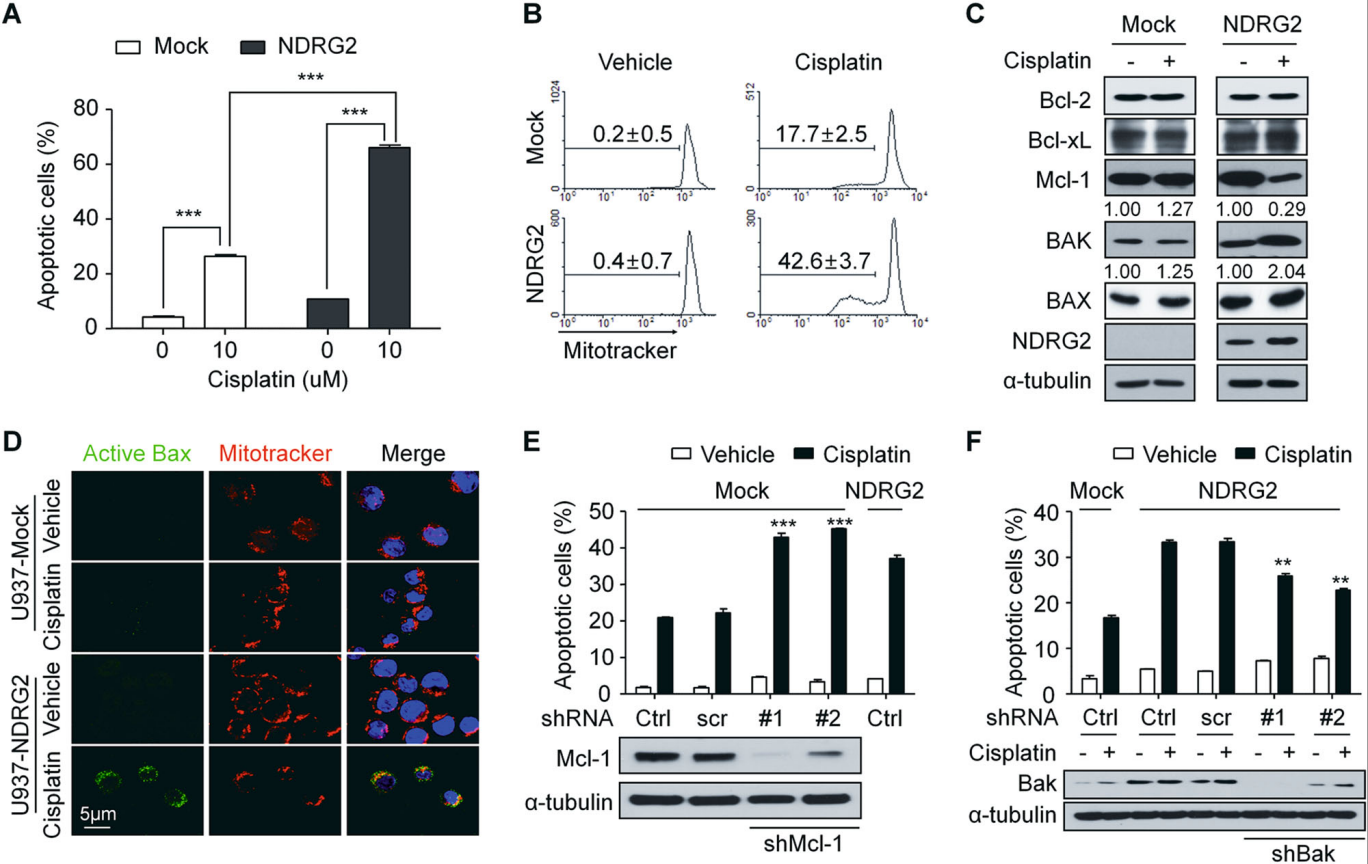
NDRG2 sensitized U937 cells to cisplatin through the modulation of the BAK-to-Mcl-1 ratio. **a** U937-Mock and -NDRG2 cells were incubated with cisplatin (10 μM, 20 h). The cells were stained with Annexin V/PI and analyzed by flow cytometry. **b** The cells were stained with MitoTracker Red CMXRos, and those with depolarized mitochondria were analyzed by flow cytometry. **c** Bcl-2 family proteins were analyzed by immunoblotting using the indicated antibodies. The normalized BAK or Mcl-1 band intensity with α-tubulin was acquired using Image J software to determine the fold induction. **d** U937-Mock and U937-NDRG2 cells treated with cisplatin were immunostained with anti-active Bax and analyzed using a confocal microscope. Active Bax was labeled with a FITC-conjugated secondary antibody (green), and the mitochondria and nuclei were stained with Mitotracker CMXRos (red) and DAPI (blue), respectively. U937-Mock cells infected with the shMcl-1 lentivirus (**e**) or U937-NDRG2 cells infected by the shBAK lentivirus (**f**) were treated with cisplatin and the apoptotic cells were analyzed by Annexin V/PI staining. Ctrl: vehicle treatment or not infected group. **p < 0.01, ***p < 0.005 by *t*-tests. Data are presented as mean ± SEM

To determine whether NDRG2 was involved in the sensitivity to cisplatin, we established a U937 cell line that expressed NDRG2 (U937-NDRG2). NDRG2 overexpression did not affect cell morphology, viability, and proliferation rate in U937 cells (Supplementary Fig. 1A and B). We found that compared with mock cells (U937-Mock), U937-NDRG2 showed significantly greater increase in apoptosis induced by cisplatin, as determined by Annexin V staining (Fig. 1a). Conversely, the knockdown of NDRG2 in U937-NDRG2 cells reduced their sensitivity to cisplatin (Supplementary Fig. 1C). NDRG2 increased cisplatin-induced mitochondrial membrane depolarization (Fig. 1b), which might be caused by both a decrease of Mcl-1 and an increase of BAK. The change of BAK-to-Mcl-1 ratio after cisplatin treatment was 1.00 to 0.96 for U937-Mock cells and 1.00 to 8.45 for U937-NDRG2 cells (Fig. 1c). Active Bax was also significantly increased by cisplatin treatment in U937-NDRG2 cells compared with that in U937-Mock cells (Fig. 1d). The activation of caspase-9 and caspase-3, and PARP cleavage were enhanced in U937-NDRG2 cells (Supplementary Fig. 1D) and a pan-caspase inhibitor, z-VAD-fmk, reduced the sensitivity to cisplatin (Supplementary Fig. 1E). To confirm the role of Mcl-1 and BAK in the sensitivity to cisplatin, we knocked down each gene. Knockdown of Mcl-1 increased the sensitivity to cisplatin in U937-Mock cells (Fig. 1e) and that of BAK reduced the sensitivity to cisplatin in U937-NDRG2 cells (Fig. 1f). These findings suggest that NDRG2 increases the sensitivity to cisplatin via a mitochondrial pathway regulated by the modulation of the BAK-to-Mcl-1 ratio.

### The JNK signaling pathway contributed to the sensitivity to cisplatin through Mcl-1 degradation in U937-NDRG2 cells

**Fig. 2:**
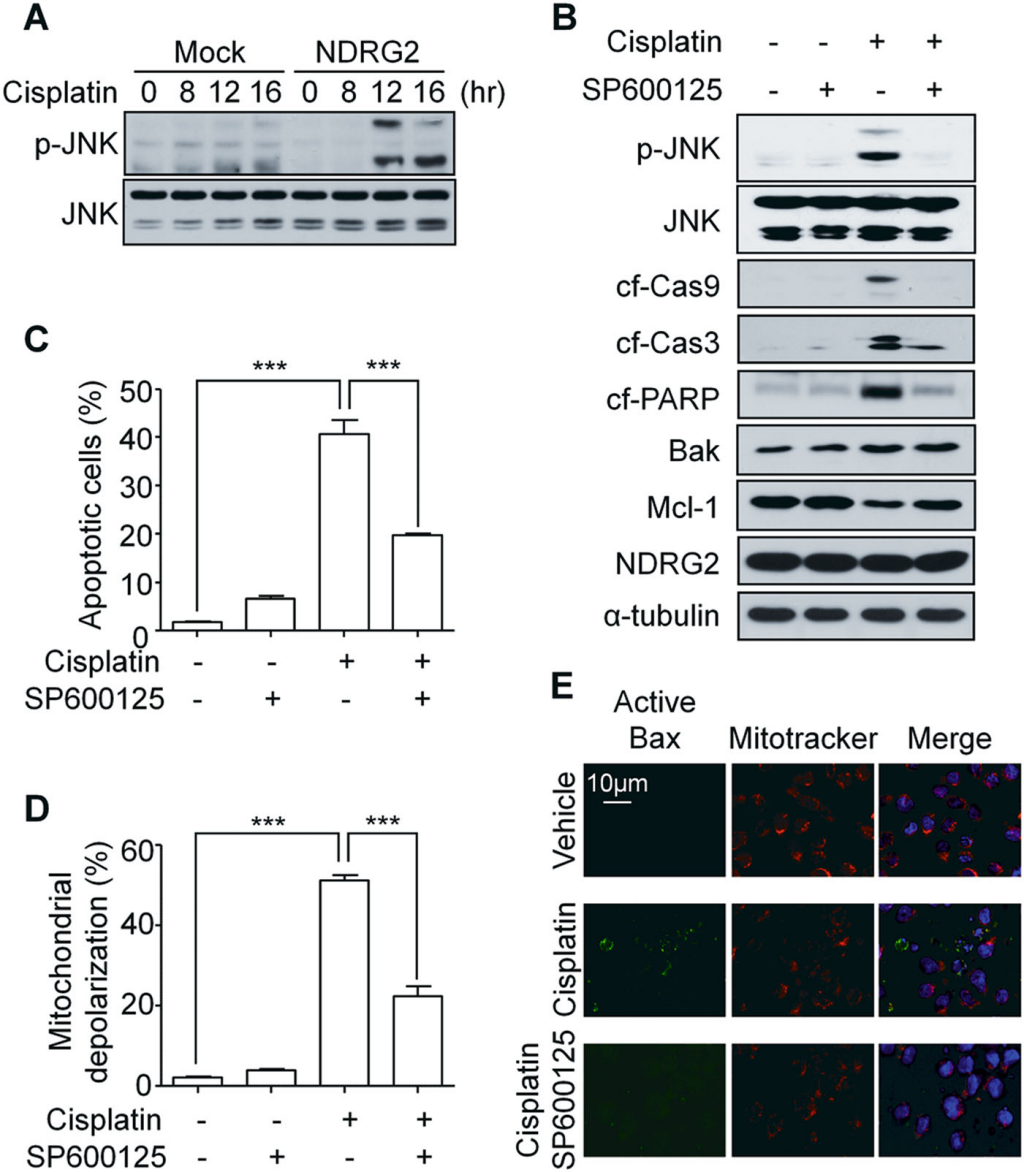
JNK activation contributed to the sensitivity of U937-NDRG2 cells to cisplatin through the induction of Mcl-1 degradation. **a** JNK phosphorylation kinetics in the cells treated with cisplatin at the indicated time points. **b** U937-NDRG2 cells were treated with cisplatin in the presence or absence of 5 μM SP600125 (a JNK inhibitor), and then the levels of p-JNK, Caspase, PARP, Mcl-1 and BAK protein were determined by immunoblotting. **c**, **d** The apoptotic cell death and mitochondrial depolarization of cisplatin-treated U937-NDRG2 cells were analyzed by flow cytometry. **e** U937-NDRG2 cells treated with cisplatin in the presence or absence of SP600125 were stained with anti-active Bax antibody (green), MitoTracker Red CMXRos (red), and DAPI (blue). The fluorescent pattern was visualized by confocal microscopy. Ctrl: vehicle-treated group. ***p < 0.005 by *t*-test. Data are presented as mean ± SEM

The decrease in Mcl-1was not regulated at the transcriptional level, but was caused by an ubiquitin-mediated degradation system that could be inhibited by MG132 (Supplementary Fig. 2). Because it has been reported that JNK activation induces ubiquitin-mediated Mcl-1 degradation^22^, we checked JNK activation in U937-NDRG2 and U937-Mock cell lysates prepared after cisplatin treatment (Fig. 2a) and observed an enhanced JNK phosphorylation (p-JNK) in U937-NDRG2 cells. To confirm whether the JNK activation was directly related to the increased sensitivity to cisplatin, U937-NDRG2 cells were treated with a JNK inhibitor, SP600125. The inhibitor inhibited the degradation of Mcl-1 but not affect BAK level (Fig. 2b). Cell death, mitochondrial membrane depolarization, and Bax activation induced by cisplatin were also inhibited by the JNK inhibitor in U937-NDRG2 cells (Fig. 2c–e). Thus, The overexpression of NDRG2 resulted in JNK activation to induce Mcl-1 degradation after cisplatin treatment, which increased the sensitivity to cisplatin.

### The level of BAK protein was regulated via the eIF2α/p-eIF2α pathway

**Fig. 3:**
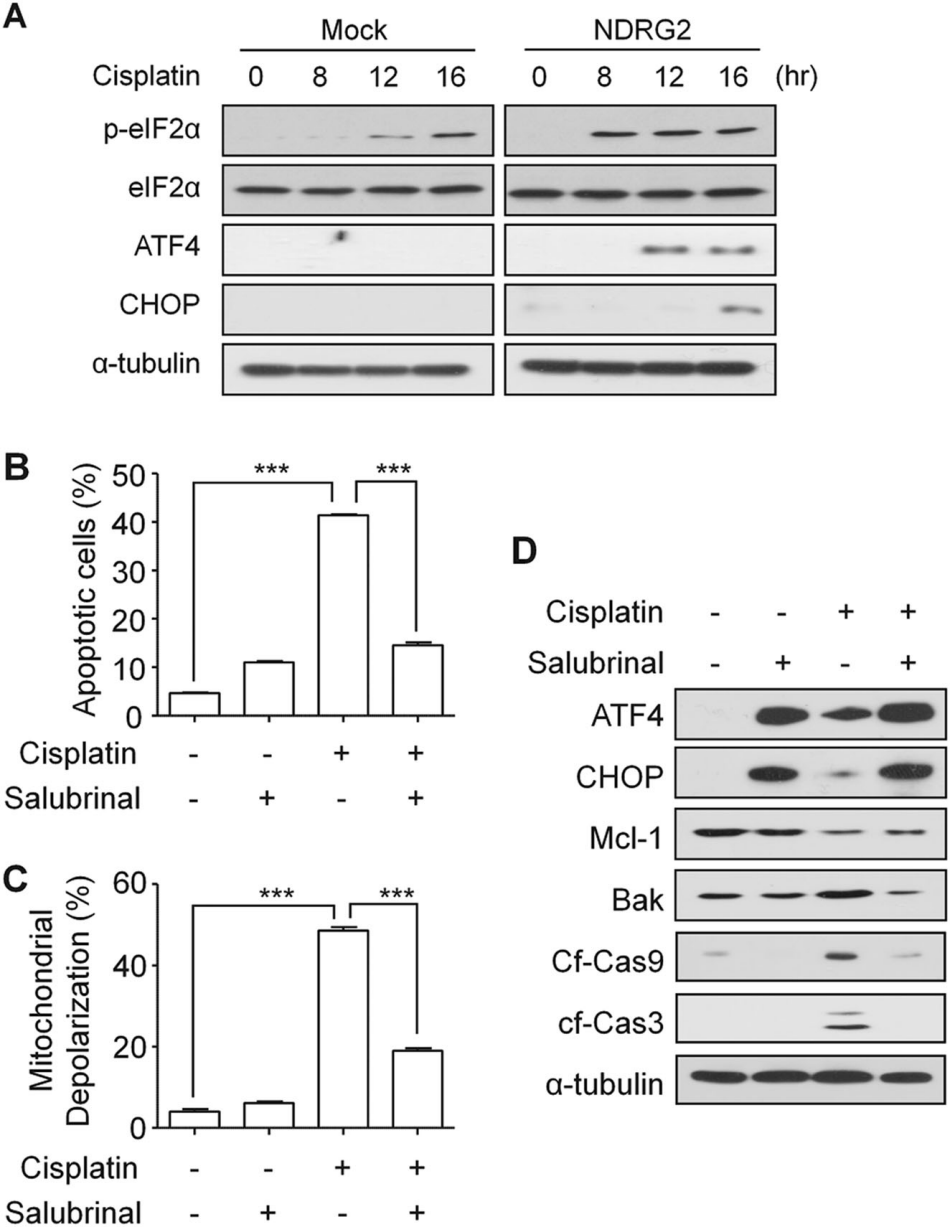
The eIF2α/p-eIF2α pathway was involved in the upregulation of BAK in U937-NDRG2 cells treated with cisplatin. **a** U937-Mock and U937-NDRG2 cells were treated with cisplatin for the indicated time, and the levels of p-eIF2α (Ser51), ATF4, and CHOP proteins were determined by immunoblotting. **b**, **c** U937-NDRG2 cells were incubated with cisplatin in the presence or absence of salubrinal (40 μM). Apoptosis and depolarization of the mitochondrial membrane were analyzed using flow cytometry. **d** The levels of the indicated proteins were determined by immunoblotting. ***p < 0.005 by *t*-test. Data are presented as mean ± SEM

To test whether ER-stress was associated with a higher sensitivity of U937-NDRG2 to cisplatin, the levels of ER-stress marker proteins, phospho-eIF2α, ATF4, and CHOP, were measured using western blotting. The significant increases in these proteins were observed in the NDRG2-U937 cells (Fig. 3a). To confirm that cisplatin induced ER-stress in U937-NDRG2 cells, cleavage of XBP-1 RNA was evaluated using RT-PCR analysis. Thapsigargin, a representative ER-stress inducer, induced the cleavage of XBP-1 transcript, but cisplatin did not do in U937-NDRG2 cells (Supplementary Fig. 3a). Furthermore, treatment with a chemical chaperone, 4-PBA, which reduces ER-stress or with BAPTA-AM, an intracellular calcium chelator that captures Ca^2+^ released from ER, did not inhibit the sensitivity of U937-NDRG2 cells to cisplatin (Supplementary Fig. 3B and C). Treatment with salubrinal, an inhibitor of eIF2α dephosphorylation, inhibited apoptosis (Fig. 3b) and the depolarization of the mitochondrial membrane (Fig. 3c) induced by ciplatin. The inhibition of eIF2α dephosphorylation led to a decrease in the level of BAK without affecting the level of Mcl-1 (Fig. 3d). These results suggest that a specific pathway that regulates eIF2α/p-eIF2α, but not ER-stress, regulates the level of BAK protein, affecting the sensitivity of U937-NDRG2 cells to cisplatin.

### The sensitivity to cisplatin mediated by NDRG2 was dependent on PKR activation

**Fig. 4:**
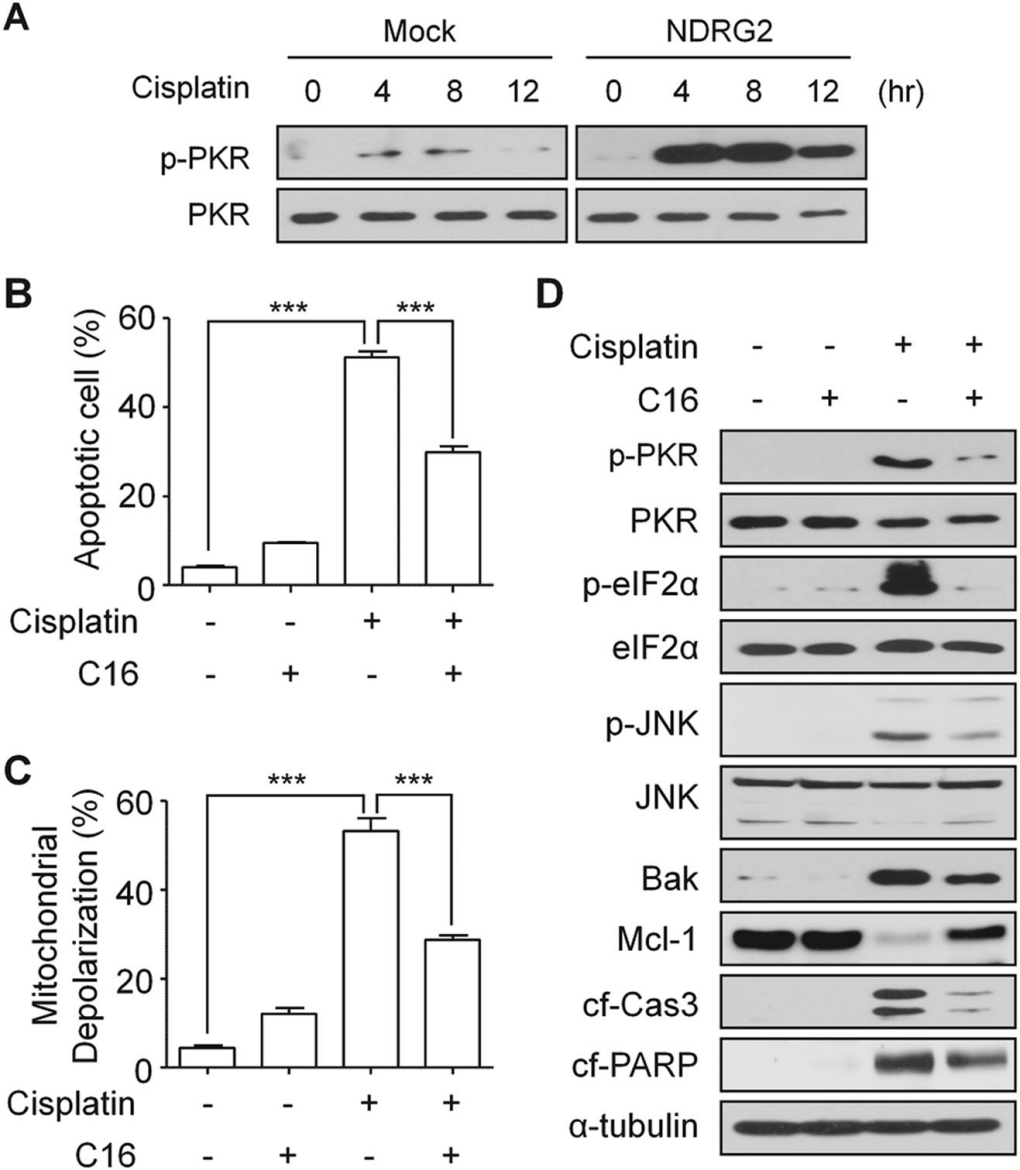
Activation of PKR was necessary for the sensitivity of U937-NDRG2 cells to cisplatin. **a** The phosphorylation of PKR was determined by immunoboltting in U937-Mock and U937-NDRG2 cells treated with cisplatin. **b**, **c** U937-NDRG2 cells were treated with cisplatin in the presence or absence of the PKR inhibitor C16 (250 nM). Apoptosis and mitochondrial potential were analyzed using flow cytometry. **d** Levels of the indicated proteins were determined by immunoblotting. ***p < 0.005 by *t*-test. Data are presented as mean ± SEM

PKR is known to be a kinase that phosphorylates both eIF2α and JNK^23^. Therefore, we confirmed that PKR activation was induced by cisplatin in U937-NDRG2 cells. Activation of PKR (p-PKR) was induced to a significantly great extent in U937-NDRG2 cells than in U937-Mock cells by cisplatin treatment (Fig. 4a). A specific inhibitor of PKR, C16, attenuated apoptosis (Fig. 4b) and the the mitochondrial membrane depolarization (Fig. 4c). Inhibiting PKR attenuated both the phosphorylation of eIF2α related to BAK induction and activation of JNK related to Mcl-1 degradation (Fig. 4d). Taken together, these findings suggest that NDRG2 mediated the higher sensitivity to cisplatin through the PKR activation pathway.

### ROS were necessary for PKR activation in U937-NDRG2 cells treated with cisplatin

**Fig. 5:**
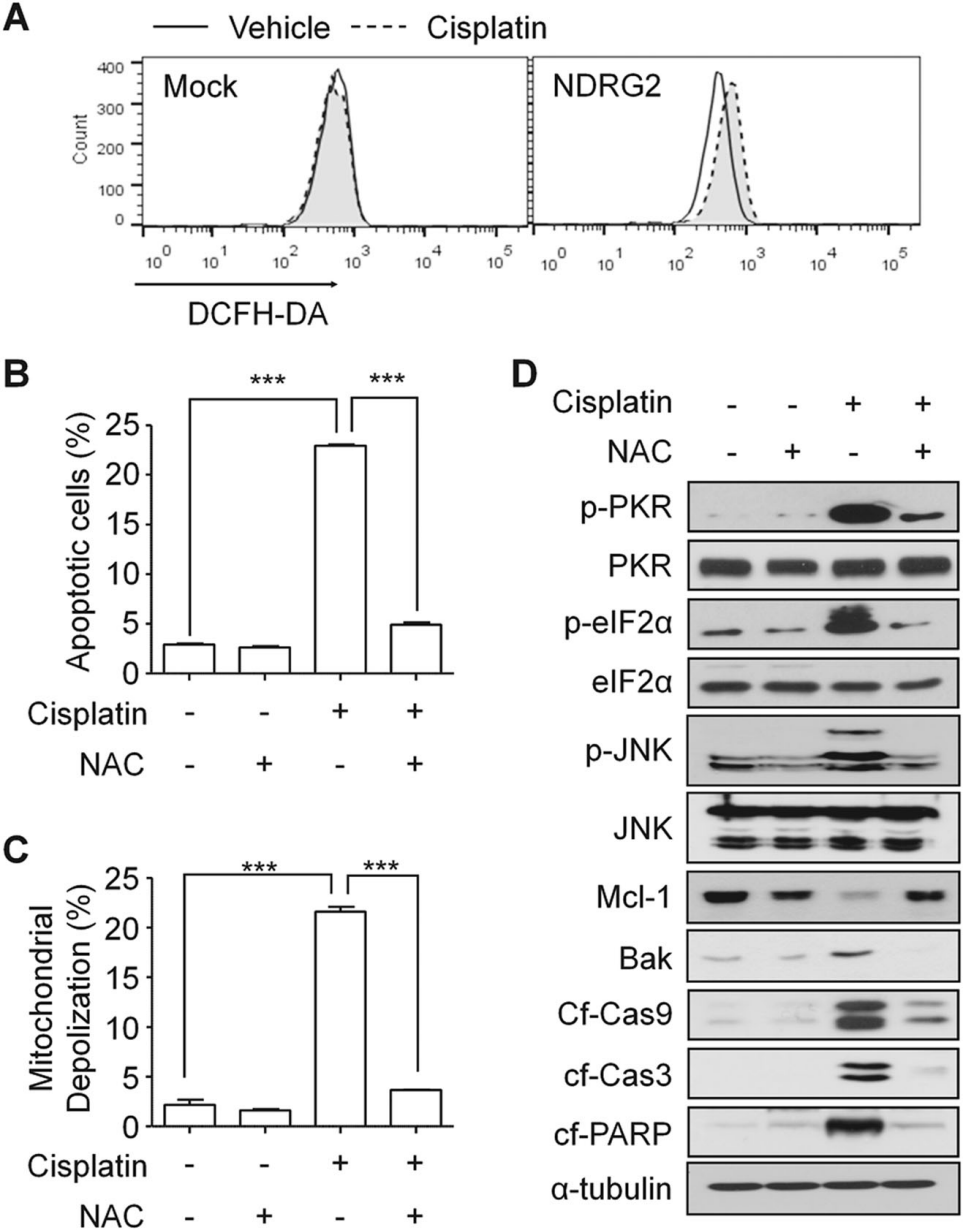
ROS produced in cisplatin-treated U937-NDRG2 cells induced PKR activation. **a** U937-Mock and U937-NDRG2 cells were treated with cisplatin, and then the level of ROS was determined using DCFH-DA staining. **b**, **c** U937-NDRG2 cells were treated with cisplatin in the presence or absence of a ROS scavenger, NAC (10 μM). Apoptosis and the mitochondrial potential were analyzed using flow cytometry. **d** Levels of the indicated proteins were determined by immunoblotting. Ctrl: vehicle-treated group. ***p < 0.005 by *t*-test. Data are presented as mean ± SEM

Among the various stresses conditions that induce PKR activation, ROS may be an important factor in apoptosis associated with PKR-mediated JNK activation and eIF2α phosphorylation^24^. Therefore, we measured ROS levels in U937-Mock and U937-NDRG2 cells treated with cisplatin. U937-NDRG2 cells exhibited higher ROS production than U937-Mock cells (Fig. 5a). Treatment with *N*-acetyl-l-cysteine (NAC), an ROS scavenger, significantly inhibited apoptosis (Fig. 5b) and mitochondrial membrane depolarization (Fig. 5c). The scavenging of ROS effectively inhibited the levels of p-PKR, p-eIF2α, and p-JNK. The decrease in Mcl-1 and increase in BAK were also inhibited; this was followed by attenuation of caspase-3 and -9 activation, and PARP cleavage (Fig. 5d). These results suggest that NDRG2-overexpression induces higher production of ROS further inducing PKR activation and triggering the modulation of the BAK-to-Mcl-1 ratio to make the cells more sensitive to cisplatin.

### NOX5 was essential for NDRG2-mediated sensitivity to cisplatin

**Fig. 6:**
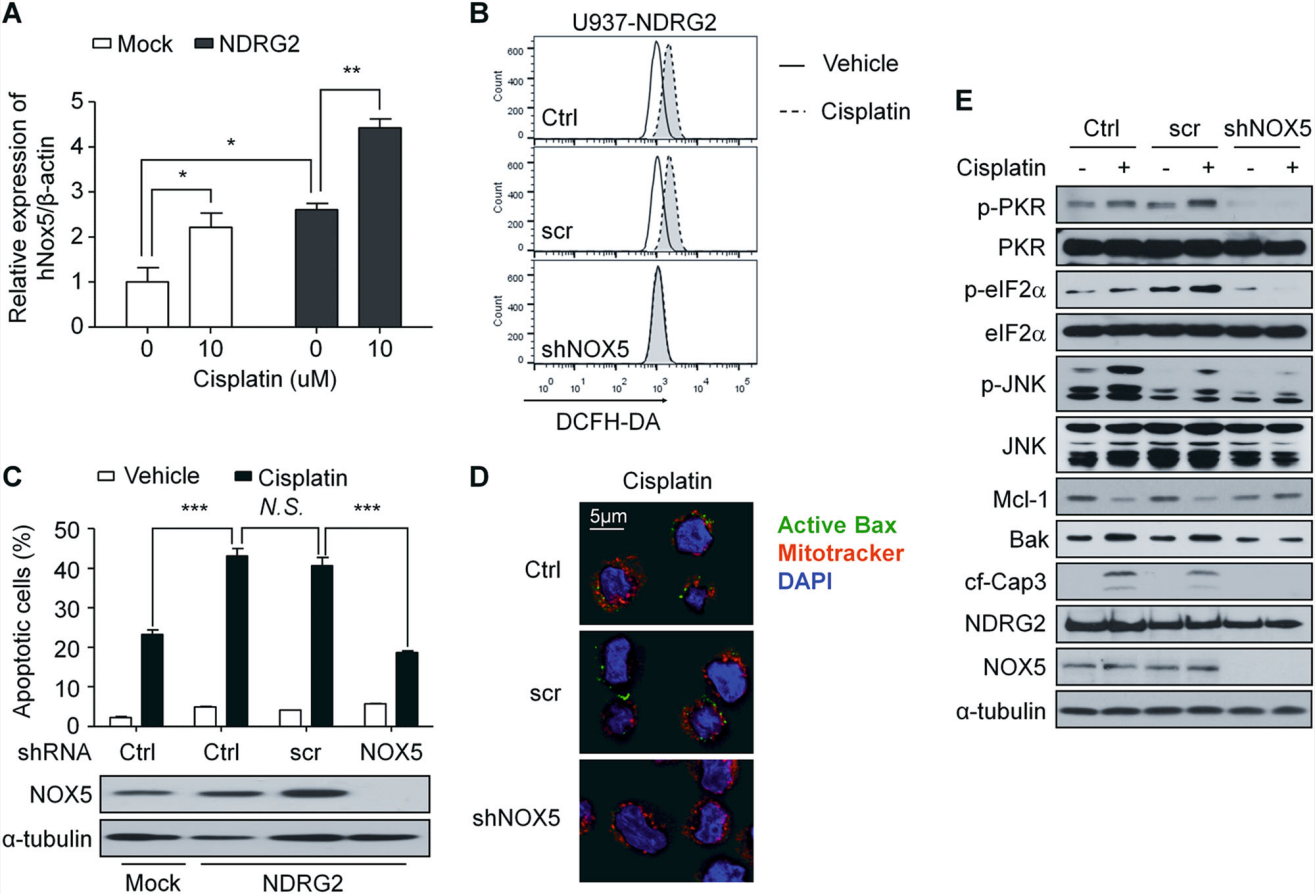
The upregulation of *NOX5* expression was essential for the higher sensitivity of U937-NDRG2 cells to cisplatin. **a** Human NOX5 expression was confirmed by quantitative RT-PCR. **b** U937-NDRG2 cells were transfected with scramble- or shNOX5-lentivirus. The knockdown efficiency of NOX5 expression was confirmed by immunoblotting, and apoptotic cell death was then analyzed by Annexin V/PI staining. **c** NOX5-knockdown cells were stained with DCFH-DA and ROS levels were determined by flow cytometry. **d** Active Bax (green) in the indicated experimental groups was visualized by confocal microscopy. U937-NDRG2 cells were treated with cisplatin for 12 h. **e** Levels of the indicated proteins were determined by immunoblotting. Ctrl: vehicle treatment or not infected group. ***p < 0.005 by *t*-test. Data are presented as mean ± SEM

**Fig. 7:**
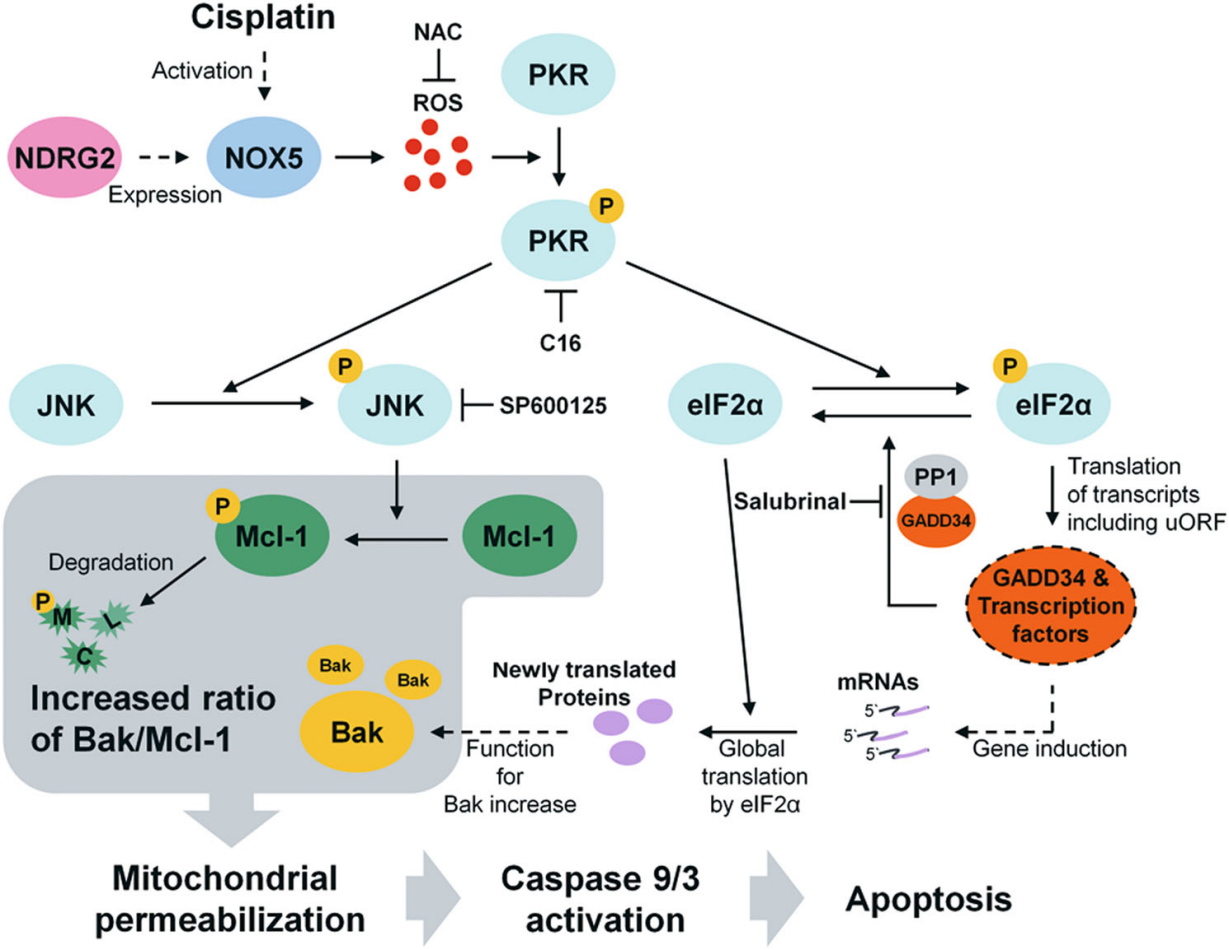
Diagrammatic representation of the proposed apoptotic pathway that mediates the sensitivity to cisplatin in NDRG2-expressing U937 cells

To identify which gene or genes regulated ROS production in U937-NDRG2 cells, we screened the expression levels of the genes related to ROS production (Supplementary Fig. 4). Among these genes, the NOX5 transcript that was increased in U937-NDRG2 cells increased to a greater extent after cisplatin treatment (Fig. 6a). Although the basal level of NOX5 was higher in U937-NDRG2 cells than that in U937-Mock cells, ROS production and apoptotic cell death in U937-NDRG2 cells seemed not to have been affected in the cisplatin-untreated group (Fig. 1a, b; Fig. 5a). To test whether NOX5 expression contributed to ROS production, we measured ROS production induced by cisplatin after NOX5-knockdown and found the ROS production to have been significantly inhibited (Fig. 6b). Apoptosis (Fig. 6c) and Bax activation (Fig. 6d) were also attenuated in NOX5-knockdown U937-NDRG2 cells. The levels of p-PKR, p-eIF2α, and p-JNK induced by cisplatin in U937-NDRG2 cells were decreased, and the changes in BAK and Mcl-1 proteins were attenuated (Fig. 6e). These finding suggest that an environment of NDRG2-overexpression results in the expression of NOX5, which produces ROS triggering apoptotic signaling after cisplatin treatment. Finally, we propose a working model, shown in Fig. 7, that explains how the function of NDRG2 is related to the higher sensitivity to cisplatin.

## Discussion

NDRG2 is known to be a tumor-suppressive protein associated with the clinicopathological features of various tumors. The downregulation of NDRG2 expression is related to cancer development and tumor aggressiveness, including high levels of invasion and metastasis, poor prognosis, and low survival rates^3^. Low effectiveness on various treatments in NDRG2-negative tumor patients may be resulted to the poor prognosis and low survival rates in these patients. Chemotherapy is one of the principal treatments for tumor patients, and its effectiveness can depend on the sensitivity of the tumor to the drug administered. The present study focused on tumor cell sensitivity to the anti-cancer drug, cisplatin, and how this was affected by NDRG2 expression. The sensitivity of tumor cells to anti-cancer drugs could be determined by drug-mediated cell death. Therefore, strategy should be developed to increase the sensitivity of tumor cells to anti-cancer drugs for improving the prognosis and survival rates of tumor patients.

The effect of NDRG2 on cell death is controversial, with reports of two opposing functions. NDRG2 demonstrated an anti-apoptotic function in focal cerebral ischemia^25^ and contributed to hypoxia-mediated radioresistance in Hela cell^26^. Conversely, it has shown a pro-apoptotic function in squamous cell carcinoma^16^, breast cancer cell^12^, hepatocarcinoma^15^, and other cancers. Although the molecular mechanism of NDRG2 in apoptosis has not been sufficiently explored, it has been shown that p53 increases NDRG2 expression and that NDRG2 is involved in p53-mediated apoptosis^14,15,27^. In this study, we used a U937 cell line having loss-of-function mutation in p53 to investigate how NDRG2 contributed to the sensitivity to cisplatin in conditions that excluded the p53-mediated apoptosis pathway induced by cisplatin.

Our main finding was that the upregulated NOX5 expression played an important role in increasing the sensitivity of cells to cisplatin via the modulation of the BAK-to-Mcl-1 ratio in U937-NDRG2 cells. This was demonstrated by the following findings. (I) NDRG2 made U937 cells more sensitive to cisplatin through the modulation of the BAK-to-Mcl-1 ratio. (II) NDRG2 induced prolonged JNK activation associated with Mcl-1 degradation under cisplatin treatment. (III) Modulation of eIF2α phosphorylation/dephosphorylation pathway was involved in the regulation of the level of BAK in U937-NDRG2 cells. (IV) PKR activation was necessary for the increased sensitivity of U937-NDRG2 cells to cisplatin. (V) ROS were necessary for PKR activation, inducing JNK activation and the modulation of the eIF2α phosphorylation/dephosphorylation pathway in U937-NDRG2 cells treated with cisplatin. (VI) The induction of NOX5 gene expression in U937-NDRG2 cells was required for the induction of ROS to increase the sensitivity to cisplatin.

Among the genes that regulate ROS production, NOX5 was upregulated in U937-NDRG2 cells compared with that in U937-Mock cells. NOXs are the enzyme family having primary function producing ROS^28,29^. The upregulation of NOX5 has been associated with several diseases, including atherosclerosis, acute myocardial infarction, aneurysms, and hypertension^30–32^. Although there are a few reports stating that NOX5 function is associated with drug-mediated cell death, NOX5 expression determined the sensitivity to cisplatin induced by ROS in U937-NDRG2 cells treated with cisplatin. Scavenging ROS or the knockdown of NOX5 successfully inhibited the decrease in Mcl-1 and increase in BAK, diminishing the sensitivity to cislatin shown by U937-NDRG2 cells. This sensitivity was regulated by the levels of Mcl-1 and BAK proteins. The modulation of the BAK-to-Mcl-1 ratio was regulated by JNK activation and the eIF2α/p-eIF2α pathway which is independent on ER-stress; these regulate Mcl-1 degradation and increase BAK, respectively. JNK phosphorylates Mcl-1 at Thr^163^, and this determines the degradation of Mcl-1 protein^33^. Salubrinal, a specific inhibitor of eIF2α/p-eIF2α pathway, inhibited the induction of BAK and attenuated the sensitivity of U937-NDRG2 cells to cisplatin. Therefore, we focused on establishing which molecule could bridge NOX5-ROS, JNK, and eIF2α/p-eIF2α pathways. Although ER-stress activates JNK and the eIF2α/p-eIF2α pathway, chemical chaperone (4-PBA) and a calcium chelator (BAPTA-AM) did not inhibit the apoptosis of U937-NDRG2 cells induced by cisplatin, and cleavage of XBP-1 transcript^34^, a representative ER-stress phenotype, was not observed. PKR is activated by various stresses, including dsRNA^35^, anti-cancer drugs^23^, and ROS^24^. The activated PKR induces phosphorylation of eIF2α^36^ and activation of JNK^23^. Although imidazolo-oxindole PKR inhibitor C16 (C16) showed only a partial inhibitory effect on apoptosis in this study, it effectively inhibited the phosphorylation of eIF2α and activation of JNK. The partial inhibitory effect of C16 may have been due to cytotoxicity which the inhibitor has by itself. Although the inhibition of p-eIF2α dephosphorylation using salubrinal and the inhibition of PKR-mediated eIF2a phosphorylation (p-eIF2a) using C16 attenuated the increase in Bak levels, each inhibition might seem to be completely opposite in terms of eIF2α regulation. Therefore, we suggest that the Bak protein level are not regulated directly by eIF2α, but might be regulated by any newly translated protein through the pathway eIF2α/p-eIF2α/eIF2α as suggested in Fig. 7.

The function of PKR in cancer is controversial^37^. PKR is known to be a p53-target gene that plays a role in the tumor-suppressive function of p53^38^. Furthermore, PKR can activate the intrinsic and extrinsic apoptotic pathways induced by various stimuli, including cancer drugs.^23,38–40^. However, it has been reported that the overexpression of PKR is associated with malignancy in many tumor tissues^41,42^ and that the function of PKR in tumorigenesis is associated of NFkB activation^43,44^. The results of the present study suggested that PKR plays a role in increasing the sensitivity of U937-NDRG2 cells, which has loss-of-function mutation in p53, to cisplatin. Although NDRG2 did not affect the PKR expression level, PKR was effectively activated by NOX5-induced ROS in U937-NDRG2 treated with cisplatin. NDRG2 may also inhibit the tumorigenic function of PKR because it can suppress NFκB activation^45,46^.

Taken together, our study demonstrated that NDRG2 was implicated in increased sensitivity to cisplatin, which was associated with inducing apoptosis. Our findings provide insights into the function of NDRG2 as a tumor suppressor and suggest that it provides a molecular target for clinical tumor therapies.

## Materials and methods

### Cell culture, reagents, and shRNAs

U937, U937-Mock, and U937-NDRG2 cell lines were cultured in RPMI1640 medium containing 10% fetal bovine serum, HEPES, β-mercaptoethanol, penicillin, and streptomycin. 293T/17 was purchased from ATCC and cultured in Dulbecco’s modified Eagle’s medium containing 10% fetal bovine serum, penicillin, and streptomycin. The cells were maintained at 37 °C in 5% CO_2_. In some of the experiments, cells were treated with chemicals such as MG132, 4-PBA, C16, DCFH-DA, *N*-acetylcysteine, thapsigargin (Sigma-Aldrich, MO, USA), z-VAD-fmk (ENZO, Seoul, Korea), and cisplatin (ILDONG Pharmaceuticals, Seoul, Korea). The JNK inhibitor, SP600125, was purchased from (Invitrogen, CA, USA). pLKO.1 based shNDRG2 (TRCN0000183898), shNOX5 (TRCN0000046098), shMcl-1 (TRCN0000005514), and shBAK (TRCN0000033466) for knockdown of the indicated genes were purchased from Sigma-Aldrich.

### Western blotting

Cell lysate samples were prepared in ProNA^TM^ CETi Lysis Buffer (TransLab, Korea). The protein extracts were concentrated by Bradford Protein assay (Bio-Rad, CA, USA). Equal amounts of the proteins were separated by SDS-PAGE and transferred onto a PVDF membrane. Membrane blocking was performed by using 5% skimmed milk (in TBS-T) for 1 h at room temperature (RT), and 30–50 μg of protein extract was used for western blotting with antibodies against Bcl-2, Bcl-xL, Bax, eIF2α, NDRG2, p-PKR (T451), PKR, NOX5 (Santa Cruz Biotechnology, TX, USA), Mcl-1, BAK, Caspase-3, Caspase-9, PARP, p-JNK (Thr183/Tyr185), JNK, p-eIF2α (ser51), ATF4, CHOP, mouse anti-rabbit IgG mAb HRP (Cell Signaling Technology, MA, USA), α-tubulin (Sigma-Aldrich, MO, USA), active-Bax (BD Bioscience, NJ, USA), and anti-mouse Ig HRP (eBioscience, CA, USA). The antibodies were treated at 4 °C for O/N and the HRP-conjugated antibodies were then treated at RT for 1 h. The HRP signal was visualized by using Clarity™ ECL Western Blotting Substrate (Bio-Rad). Band intensity was calculated with Image J software.

### Lentivirus production and infection

For the knockdown of NDRG2, Mcl-1, BAK, and NOX5, the validated shRNAs against these genes were purchased from Sigma-Aldrich, and the viral production and infection of the cell lines were performed according to the ViraPower^TM^ Lentiviral Expression System protocol (Invitrogen, CA, USA). Virus-containing supernatant was harvested and stored at −80 °C. The supernatant was used to treat U937 cells in a 12-well plate and was selected using 5 μg/ml of puromycin for O/N.

### Flow cytometry

For the analysis of apoptosis, cells were stained with 1× Annexin V binding buffer containing Annexin V-FITC (BD Bioscience, NJ, USA) and propidium iodide (PI; Sigma-Aldrich). For the analysis of mitochondrial potential, cells were stained with 250 nM Mitotracker CMXRos (Invitrogen, CA, USA) for 30 min at 37 °C. For the analysis of ROS production, cells were treated with 20 μM DCFH-DA. Fluorescence was measured with a FACSVerse™ flow cytometer (BD Bioscience, NJ, USA) and analyzed with FlowJo V10 software (FlowJo, OR, USA).

### Confocal microscopy

The cells were fixed with 4% paraformaldehyde for 30 min at RT and then permeabilized with 0.1% trioton X-100 in PBS for 15 min at RT. The cells were treated with active-Bax antibody (1:50 in PBS) antibody was treated for O/N at 4 °C and then α-Mouse-FITC (1:100 in PBS) for 2 h at 4 °C. They were then treated with 40 μg/ml of DAPI and washed five times with PBS. Coverslips were mounted on a slideglass with mounting solution, and the fluorescence was visualized with an Olympus FluoView™ FV1000 Confocal Microscope (Olympus, Tokyo, Japan).

### Reverse transcription and real-time polymerase chain reaction

Total RNA was extracted from cells using RiboEx^TM^ (GeneAll, Seoul, Korea), and cDNA was synthesized using a reverse transcription kit (Thermo Fisher Scientific, MA, USA). Reverse transcription polymerase chain reaction (RT-PCR) analysis was performed using the DNA Engine Dyad^®^ Peltier Thermal Cycler (Bio-Rad). Real-time PCR was performed using SSoFast™ EvaGreen^®^ Supermix and a CFX96™ Real-Time PCR Detection System (Bio-Rad). The primers for PCR are listed in Supplementary Table 1.

### Statistics

The experimental data were analyzed using unpaired Student’s *t*-test. A *p* value < 0.05 was considered statistically significant.

## Electronic supplementary material

Supplemental material

## References

[1] Hu W (2016). Clinical and pathological significance of N-Myc downstream-regulated gene 2 (NDRG2) in diverse human cancers. Apoptosis.

[2] Oh SS (2012). NDRG2 correlated with favorable recurrence-free survival inhibits metastasis of mouse breast cancer cells via attenuation of active TGF-beta production. Carcinogenesis.

[3] Hu W (2016). Emerging role of N-myc downstream-regulated gene 2 (NDRG2) in cancer. Oncotarget.

[4] Yao L, Zhang J, Liu X (2008). NDRG2: a Myc-repressed gene involved in cancer and cell stress. Acta Biochim. Biophys. Sin..

[5] Deng Y (2003). N-Myc downstream-regulated gene 2 (NDRG2) inhibits glioblastoma cell proliferation. Int. J. Cancer.

[6] Lee DC (2008). Functional and clinical evidence for NDRG2 as a candidate suppressor of liver cancer metastasis. Cancer Res..

[7] Siddik ZH (2003). Cisplatin: mode of cytotoxic action and molecular basis of resistance. Oncogene.

[8] Miyashita T, Reed JC (1995). Tumor suppressor p53 is a direct transcriptional activator of the human bax gene. Cell.

[9] Oda E (2000). Noxa, a BH3-only member of the Bcl-2 family and candidate mediator of p53-induced apoptosis. Science.

[10] Yu J, Zhang L, Hwang PM, Kinzler KW, Vogelstein B (2001). PUMA induces the rapid apoptosis of colorectal cancer cells. Mol. Cell.

[11] Harn HJ (1996). Down regulation of bcl-2 by p53 in nasopharyngeal carcinoma and lack of detection of its specific t(14;18) chromosomal translocation in fixed tissues. Histopathology.

[12] Kim HS (2014). NDRG2 overexpression enhances glucose deprivation-mediated apoptosis in breast cancer cells via inhibition of the LKB1-AMPK pathway. Genes Cancer.

[13] Wei Y (2017). NDRG2 promotes adriamycin sensitivity through a Bad/p53 complex at the mitochondria in breast cancer. Oncotarget.

[14] Liu N (2008). N-Myc downstream-regulated gene 2 is involved in p53-mediated apoptosis. Nucleic Acids Res..

[15] Cao W (2014). The effect of adenovirus-conjugated NDRG2 on p53-mediated apoptosis of hepatocarcinoma cells through attenuation of nucleotide excision repair capacity. Biomaterials.

[16] Wang S (2015). Adenovirus siMDM2 and NDRG2 gene therapy inhibits cell proliferation and induces apoptosis of squamous cell carcinoma. Cell Biochem. Biophys..

[17] Scheffner M, Huibregtse JM, Vierstra RD, Howley PM (1993). The HPV-16 E6 and E6-AP complex functions as a ubiquitin-protein ligase in the ubiquitination of p53. Cell.

[18] Camus S (2007). Ubiquitin-independent degradation of p53 mediated by high-risk human papillomavirus protein E6. Oncogene.

[19] Thomas MC, Chiang CM (2005). E6 oncoprotein represses p53-dependent gene activation via inhibition of protein acetylation independently of inducing p53 degradation. Mol. Cell.

[20] Liu J (2012). Knockdown of NDRG2 sensitizes cervical cancer Hela cells to cisplatin through suppressing Bcl-2 expression. BMC Cancer.

[21] Sugimoto K (1992). Frequent mutations in the p53 gene in human myeloid leukemia cell lines. Blood.

[22] Inuzuka H (2011). SCF(FBW7) regulates cellular apoptosis by targeting MCL1 for ubiquitylation and destruction. Nature.

[23] Peidis P, Papadakis AI, Muaddi H, Richard S, Koromilas AE (2011). Doxorubicin bypasses the cytoprotective effects of eIF2alpha phosphorylation and promotes PKR-mediated cell death. Cell Death Differ..

[24] Pyo CW, Lee SH, Choi SY (2008). Oxidative stress induces PKR-dependent apoptosis via IFN-gamma activation signaling in Jurkat T cells. Biochem. Biophys. Res. Commun..

[25] Wang F (2013). NDRG2 is involved in anti-apoptosis induced by electroacupuncture pretreatment after focal cerebral ischemia in rats. Neurol. Res..

[26] Liu J (2010). HIF-1 and NDRG2 contribute to hypoxia-induced radioresistance of cervical cancer Hela cells. Exp. Cell Res..

[27] Li Y (2013). NDRG2 is a novel p53-associated regulator of apoptosis in C6-originated astrocytes exposed to oxygen-glucose deprivation. PLoS ONE.

[28] Drummond GR, Selemidis S, Griendling KK, Sobey CG (2011). Combating oxidative stress in vascular disease: NADPH oxidases as therapeutic targets. Nat. Rev. Drug Discov..

[29] Lassegue B, San Martin A, Griendling KK (2012). Biochemistry, physiology, and pathophysiology of NADPH oxidases in the cardiovascular system. Circ. Res..

[30] Yu P (2014). Unique role of NADPH oxidase 5 in oxidative stress in human renal proximal tubule cells. Redox Biol..

[31] Manea A (2015). Human monocytes and macrophages express NADPH oxidase 5; a potential source of reactive oxygen species in atherosclerosis. Biochem. Biophys. Res. Commun..

[32] Holterman CE (2014). Nephropathy and elevated BP in mice with podocyte-specific NADPH oxidase 5 expression. J. Am. Soc. Nephrol..

[33] Inoshita S (2002). Phosphorylation and inactivation of myeloid cell leukemia 1 by JNK in response to oxidative stress. J. Biol. Chem..

[34] Calfon M (2002). IRE1 couples endoplasmic reticulum load to secretory capacity by processing the XBP-1 mRNA. Nature.

[35] Patel RC, Sen GC (1994). Characterization of the interactions between double-stranded RNA and the double-stranded RNA binding domain of the interferon induced protein kinase. Cell Mol. Biol. Res..

[36] Donnelly N, Gorman AM, Gupta S, Samali A (2013). The eIF2alpha kinases: their structures and functions. Cell Mol. life Sci..

[37] Marchal JA (2014). The impact of PKR activation: from neurodegeneration to cancer. FASEB J..

[38] Yoon CH, Lee ES, Lim DS, Bae YS (2009). PKR, a p53 target gene, plays a crucial role in the tumor-suppressor function of p53. Proc. Natl Acad. Sci. USA.

[39] Garcia MA, Guerra S, Gil J, Jimenez V, Esteban M (2002). Anti-apoptotic and oncogenic properties of the dsRNA-binding protein of vaccinia virus, E3L. Oncogene.

[40] Garcia MA (2011). The chemotherapeutic drug 5-fluorouracil promotes PKR-mediated apoptosis in a p53-independent manner in colon and breast cancer cells. PLoS ONE.

[41] Hiasa Y (2003). Protein kinase R is increased and is functional in hepatitis C virus-related hepatocellular carcinoma. Am. J. Gastroenterol..

[42] Kim SH, Gunnery S, Choe JK, Mathews MB (2002). Neoplastic progression in melanoma and colon cancer is associated with increased expression and activity of the interferon-inducible protein kinase, PKR. Oncogene.

[43] Prasad S, Ravindran J, Aggarwal BB (2010). NF-kappaB and cancer: how intimate is this relationship. Mol. Cell Biochem..

[44] Delgado Andre N, De Lucca FL (2007). Knockdown of PKR expression by RNAi reduces pulmonary metastatic potential of B16-F10 melanoma cells in mice: possible role of NF-kappaB. Cancer Lett..

[45] Ichikawa T, Nakahata S, Fujii M, Iha H, Morishita K (2015). Loss of NDRG2 enhanced activation of the NF-kappaB pathway by PTEN and NIK phosphorylation for ATL and other cancer development. Sci. Rep..

[46] Kim A (2009). Suppression of NF-kappaB activity by NDRG2 expression attenuates the invasive potential of highly malignant tumor cells. Carcinogenesis.

