# Supplementary material for: NDRG2 contributes to cisplatin sensitivity through modulation of BAK-to-Mcl-1 ratio
Source: Cell Death Dis. 2018 Jan 18;9(2):30. doi: 10.1038/s41419-017-0184-3 (PMC5833685; doi:10.1038/s41419-017-0184-3)
Supplement: Supplementary file 1 — Supplemental material [file 41419_2017_184_MOESM1_ESM.docx]

| Supplementary Table 1. Primer pairs for gene amplification. | | | | |
| --- | --- | --- | --- | --- |
| Gene | Forward sequences (5′ to 3′) | Reverse sequences (5′ to 3′) | PCR product length (bp) | Annealing Tm (℃) |
| *Mcl-1* | CTCTCATTTCTTTTGGTGCCT | ATTCCTGATGCCACCTTCTA | 194 | 58 |
| *b-actin* | GGTCATCACTATTGGCAACG | ACGGATGTCAACGTCACACT | 133 | 60 |
| *XBP-1* | TTACGAGAGAAAACTCATGGC | GGGTCCAAGTTGTCCAGAATGC | 257 or 283 | 58 |
| *hCAT* | TTGGCCTCACAAGGACTACC | ATCTCCGCACTTCTCCAGAA | 389 | 60 |
| *hTxn* | GCCATCTGCGTGACAATAAA | ATTGCCCCCAACAAGAGATT | 218 | 58 |
| *hSOD1* | AGGGCATCATCAATTTCGAG | ACATTGCCCAAGTCTCCAAC | 217 | 58 |
| *hSOD2* | GTTGGCCAAGGGAGATGTTA | CCTTGCAGTGGATCCTGATT | 300 | 60 |
| *hSOD3* | AGACATGTACGCCAAGGTCA | GAACTGGTGCACGTGGATG | 247 | 60 |
| *hGLRX* | GGAAGGTGGTTGTGTTCATC | CCCGTGAGCTGTTGCAAATA | 159 | 60 |
| *hGLRX2* | TTGGAGGTGCAACTGACACT | CCACCGCAATTTATTGTTCA | 252 | 56 |
| *hGPx1* | AGCCCAACTTCATGCTCTTC | GATGTCAGGCTCGATGTCAA | 260 | 60 |
| *hGPx2* | CAAGCGCCTCCTTAAAGTTG | AGAGGGTTGGGAGAGGAAAA | 203 | 60 |
| *hGPx3* | ATTCGGTCTGGTCATTCTGG | TACATGGTGGACATGGGATG | 442 | 60 |
| *hGPx4* | GCACATGGTTAACCTGGACA | TCACGCAGATCTTGCTGAAC | 254 | 60 |
| *hGPx5* | TAGCAATGGGGTCACAGTCA | AGGTGCCATAACCTGAATGC | 254 | 60 |
| *hGPx6* | CGTCCTGTTTGTCAATGTGG | CAGCGGATATCATGGACCTT | 354 | 60 |
| *hGPx7* | ACTGGTGTCGCTGGAGAAGT | GTCTGGGCCAGGTACTTGAA | 297 | 60 |
| *hPRDX1* | GTGATTGGTGCTTCTGTGGA | TGATCTGCCGAAGAATACCC | 211 | 60 |
| *hPRDX2* | TCAAAGAGGTGAAGCTGTCG | CCAGGTGGGTGAACTGAGAG | 180 | 60 |
| *hPRDX3* | GCAGATTTCCCGAGACTACG | CTCTTTGGAAGCAGCTGGAC | 250 | 62 |
| *hPRDX4* | CTCCCTGCACCTAAGCAAAG | TAATCCAGGCCAAATGGGTA | 266 | 58 |
| *hPRDX6* | TGATAGGAATCGGGAGCTTG | TCTTCTTCAGGGATGGTTGG | 279 | 60 |
| *hNOX1* | CTGTTTGTGGATGCCTTCCT | TGTGGAAGGTGAGGTTGTGA | 232 | 60 |
| *hNOX2* | GGAGTTTCAAGATGCGTGGAAACTA | GCCAGACTCAGAGTTGGAGATGCT | 550 | 64 |
| *hNOX3* | GGGCAGTACATCTTGGTGCA | ACATCTGTCAGGGCAGTTCC | 230 | 60 |
| *hNOX4* | TGCCATGAAGCAGGACTCTA | AGGGTCTCTTTGGTTTCCAG | 192 | 60 |
| *hNOX5* | GCGGTCTTTCGAGTGGTTTG | CACCTTGCCCTTCTTCTCAG | 277 | 62 |

**Supplementary Materials**

**Supplementary Fig. 1. NDRG2-mediated sensitivity to cisplatin is caspase-dependent**

(A) Cells were stained with anti-NDRG2 antibody (Red) and DAPI. Contrast and fluorescent images were visualized by confocal microscopy. (B) Cells were seeded on to six-well plates and cultured for the indicated hours. The number of cells was counted using a hemocytometer. Cell viability was also checked by MTS assay after incubation for 24hr. (C) U937-NDRG2 cells were transduced using the shNDRG2 lentivirus. The knockdown efficiency of NDRG2 was confirmed by immunoblotting, and apoptotic cell death was then analyzed by Annexin V/PI staining. (D) Caspase-3, caspase-9, and PARP cleavage were determined by immunoblotting. (E) U937-NDRG2 cells were treated with cisplatin in the presence or absence of zVad-fmk (a pan-caspase inhibitor, 50 μM), and apoptotic cell death was analyzed by Annexin V/PI staining. *, *p<* 0.05, **, *p* < 0.01 by *t*-tests. Data are presented as mean ± SEM.

**
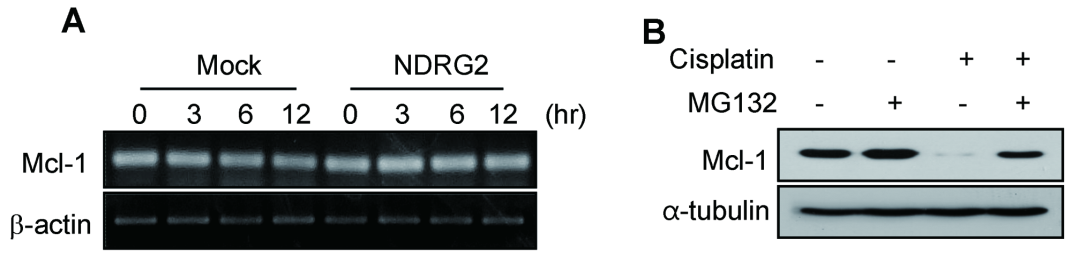
**

**Supplementary Fig. 2. Mcl-1 was degraded by ubiquitin**-poteasome system **in U937-NDRG2 cells**

(A) The Mcl-1 mRNA level was determined by real-time PCR. (B) U937-NDRG2 cells were treated with cisplatin in the presence or absence of MG132, and the levels of Mcl-1 and BAK proteins were determined by immunoblotting.

**
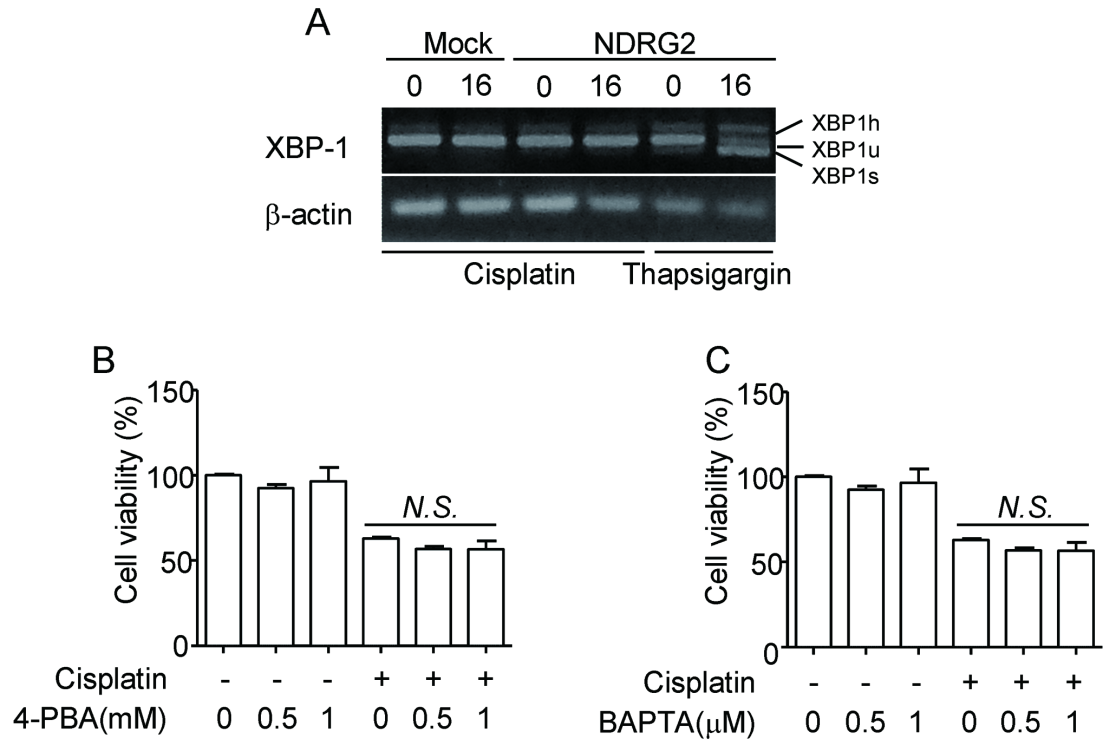
**

**Supplementary Fig. 3. Endoplasmic reticulum stress was not associated with the sensitivity to cisplatin of U937-NDRG2 cells**

(A) U937-Mock and U937-NDRG2 cells were treated with cisplatin or thapsigargin. The cleavage of the XBP transcript was determined by RT-PCR. U937-NDRG2 cells were treated with cisplatin in the presence or absence of the chemical chaperone 4-PBA (B) or Ca^2+^ chelator BAPTA-AM (C). Cell viability was determined using MTS assay.

**
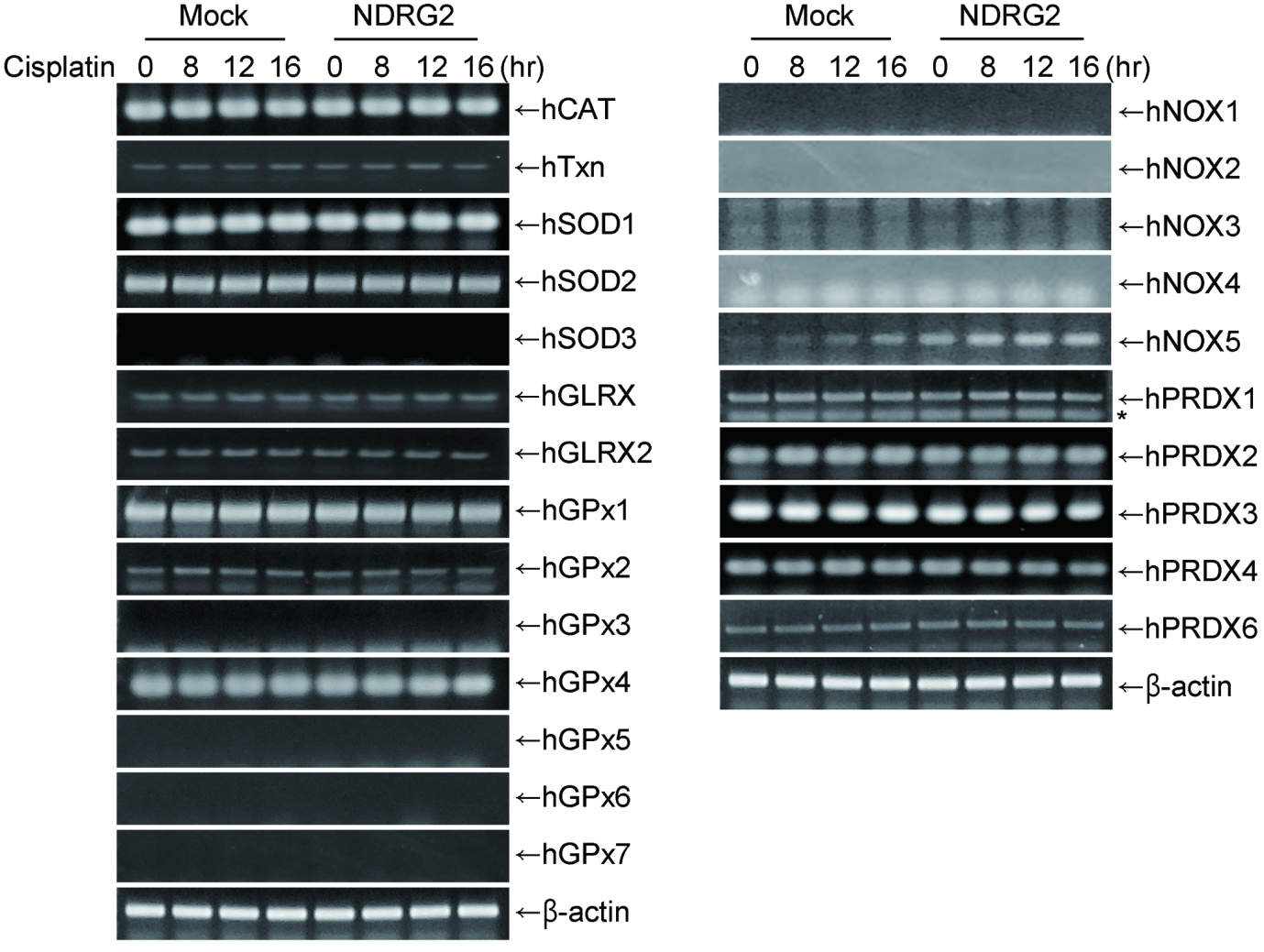
**

**Supplementary Fig. 4. The expression profiling of genes that regulate intracellular reactive oxygen species levels in U937-Mock and U937-NDRG2 cells**
